# Pompe disease in Austria: clinical, genetic and epidemiological aspects

**DOI:** 10.1007/s00415-017-8686-6

**Published:** 2017-11-27

**Authors:** W. N. Löscher, M. Huemer, T. M. Stulnig, P. Simschitz, S. Iglseder, C. Eggers, H. Moser, D. Möslinger, M. Freilinger, F. Lagler, S. Grinzinger, M. Reichhardt, R. E. Bittner, W. M. Schmidt, U. Lex, M. Brunner-Krainz, S. Quasthoff, J. V. Wanschitz

**Affiliations:** 1 Department of Neurology, Medical University Innsbruck, Anichstrasse 35, 6020 Innsbruck, Austria; 2 Department of Paediatrics, Landeskrankenhaus Bregenz, Bregenz, Austria; 3 Christian Doppler-Laboratory for Cardio-Metabolic Immunotherapy and Clinical Division of Endocrinology and Metabolism, Internal Medicine III, Medical University of Vienna, Vienna, Austria; 4 Department of Neurology, Klinikum Klagenfurt Am Wörthersee, Klagenfurt, Austria; 5 Department of Neurology, Barmherzige Brüder, Linz, Austria; 6 Neurological Therapy Center Gmundnerberg, Altmünster, Austria; 7 Department of Paediatrics and Adolescent Medicine, Medical University of Vienna, Vienna, Austria; 8 Department for Paediatrics, Institute for Inborn Errors of Metabolism, Paracelsus Medical University, Salzburg, Austria; 9 Department of Neurology, Paracelsus Medical University, Salzburg, Austria; 10 Department of Paediatrics, Kaiser-Franz-Josef-Hospital, Vienna, Austria; 11 Neuromuscular Research Department, Center of Anatomy and Cell Biology, Medical University of Vienna, Vienna, Austria; 12 Privatklinik Maria Hilf, Klagenfurt Am Wörthersee, Austria; 13 Department of Pediatrics, Medical University Graz, Graz, Austria; 14 Department of Neurology, Medical University Graz, Graz, Austria

**Keywords:** Pompe disease, Clinical phenotype, Genetics, Enzyme replacement therapy, Epidemiology

## Abstract

In this study, we performed a survey of infantile and late-onset Pompe disease (IOPD and LOPD) in Austria. Paediatric and neuromuscular centres were contacted to provide a set of anonymized clinical and genetic data of patients with IOPD and LOPD. The number of patients receiving enzyme replacement therapy (ERT) was obtained from the pharmaceutical company providing alglucosidase alfa. We found 25 patients in 24 families, 4 IOPD and 21 LOPD with a resulting prevalence of 1:350,914. The most frequent clinical manifestation in LOPD was a lower limb-girdle phenotype combined with axial weakness. Three patients were clinically pauci- or asymptomatic and were diagnosed because of persistent hyperCKemia. Diagnostic delay in LOPD was 7.4 ± 9.7 years. The most common mutation was c.-32-13T > G. All IOPD and 17 symptomatic LOPD patients are receiving ERT. Standardized follow-up was only available in six LOPD patients for the 6-min walk test (6minWT) and in ten for the forced vital capacity (FVC). Mean FVC did not decline (before ERT; 63.6 ± 39.7%; last evaluation during ERT: 61.9 ± 26.9%; *P* = 0.5) while there was a trend to decline in the mean distance covered by the 6minWT (before ERT: 373.5 ± 117.9 m; last evaluation during ERT: 308.5 ± 120.8 m; *P* = 0.077). The study shows a lower prevalence of Pompe disease in Austria than in other European countries and corroborates a limb-girdle phenotype with axial weakness as the most common clinical presentation, although asymptomatic hyperCKemia may be the first indication of LOPD.

## Introduction

Pompe disease, also known as glycogenosis type 2 or acid maltase deficiency, is an autosomal recessive disease caused by > 450 mutations in the *GAA* gene (http://cluster15.erasmusmc.nl/klgn/pompe/mutations.html) which lead to deficiency of the lysosomal enzyme acid alpha-glucosidase [1]. Depending on residual enzyme activity symptoms may develop during the first months of life (infantile-onset Pompe disease, IOPD) or during childhood, adolescence or adulthood (late-onset Pompe disease, LOPD) [2]. Untreated IOPD causes rapidly progressive muscle weakness and cardiomyopathy which usually is fatal within 1–2 years [3]. LOPD generally takes a milder course with slowly progressive muscle weakness without cardiomyopathy. However, overtime many patients need ventilatory support due to respiratory weakness [1].

Pompe disease is a rare disorder with an estimated prevalence of 1:283,000 in Europe [4]. However, it is assumed that the prevalence is higher as several studies in patients with unexplained myopathies detected new cases with undiagnosed LOPD [5, 6]. Since enzyme replacement therapy (ERT) has become the standard treatment for Pompe disease [7], increased efforts have been undertaken to further delineate the phenotype of Pompe disease to allow early diagnosis and treatment.

The efficacy of ERT is impressive in most IOPD patients, improving cardiac, respiratory and motor function as well as overall survival [8]. In LOPD, the effect of ERT, although significant in a RCT and also shown in a large meta-analysis [7, 9], is less pronounced. The high costs and the limited effects of treatment resulted in guidelines offering criteria when to start and when to stop treatment in some countries [10] and recently European guidelines were formulated [11].

To expand clinical, genetic and epidemiologic data on this rare disease and to provide results on the effects of ERT, we report the Austrian cohort of IOPD and LOPD patients.

## Methods

This retrospective observational study was approved by the ethics committee of the Medical University, Innsbruck. Austrian neuromuscular and paediatric centres were contacted and asked whether they currently manage or treat patients with IOPD or LOPD. To cross-check, we also contacted the pharmaceutical company providing ERT (Sanofi-Genzyme) to inquire about the number of Austrian patients receiving alglucosidase alfa (Myozyme^®^).

Centres were asked to provide a set of anonymized clinical and genetic data of their patients: age; gender; age at symptom onset; age at diagnosis; genetic results; biochemical data; EMG results; age when ERT was started; distribution of muscle weakness, respiratory function, 6-min walk test (6minWT) before ETR and at last visit; in IOPD cardiac function and CRIM (cross-reactive immunological material) status.

Genetic test results were compared with the Pompe mutations database (http://cluster15.erasmusmc.nl/klgn/pompe/mutations.html) and mutations in the *GAA* gene are reported based on reference sequence NM_000152.3. Results are given as mean ± SD (range).

## Results

25 patients with Pompe disease from 24 families, 4 with IOPD and 21 with LOPD, were detected. The calculated prevalence of Pompe disease in Austria is 1:350,914 (population of 8,772,865; http://www.statistik-oesterreich.at/web_de/klassifikationen/regionale_gliederungen/bundeslaender/index.html).

### IOPD

In these four patients (three female) first symptoms developed at the age of 2, 2, 9 and 10 months. Diagnosis of Pompe disease was made at 3, 6, 12 and 18 months, respectively. All displayed motor developmental delay and generalized weakness. The two younger children had the classical presentation with cardiomyopathy (CMP). All patients had normal cognitive function on formal evaluation. Only one patient underwent MRI of the brain which was normal. Evoked potentials and nerve conduction studies have not been performed.

All patients were found to be CRIM positive and ERT was initiated immediately after diagnosis. At present, these patients have been receiving ERT for 8.75, 9.5 and 11 years, without developing antibodies against rhGAA (Genzyme Clinical Specialty Lab, One Mountain Road, Framingham, MA). ERT was just recently started in the boy (c.-32-13T > G/deletion exon 18).

ERT improved CMP in both children with classical early-onset disease. The later (at 6 months) diagnosed girl (c.784G > A/c.1057delC, new mutation) had never been able to walk and showed dramatic deterioration of her muscular phenotype following a severe pulmonary infection at age 8 years. At present, she has a tracheostoma and needs ventilatory support for almost 24 h/day. The other girl (c.1195-2A > G/c.1195-2A > G) with symptom onset at age 2 months and immediate start of ERT is ambulatory and without ventilation. The girl with disease onset at 9 months without CMP (c.-32-13T > G/c.1050-1051delGG) is fully ambulatory but requires non-invasive ventilation during nighttime.

### LOPD

Selected individual clinical findings are shown in Table 1.

**Table 1:** Demographic, clinical and genetic data of the Austrian LOPD patients

ID	Gender	Age	Symptom onset [years]	Findings at presentation	Diagnostic delay [years]	Duration of ERT [years]	Genetics allel 1	Genetics allel 2
1	M	36	21	l, a	6	8	c.-32-13T > G	c.877G > A
2	F	65	44	l, a, r	10	11	c.-32-13T > G	c.1912G > T
3	F	26	18	l, a, r	1	7	c.-32-13T > G	c.2281delinsAT
4	F	58	25	l, a, r	18	8	c.-32-13T > G	c.307T > G
4a	M	56	46	r	0	6	c.-32-13T > G	c.307T > G
6	F	37	22	l, a, r	1	8	c.-32-13T > G	**c.1076-2G** **>** **A**
7	M	27	11	l, a	5	11	c.692 + 5G > T	c.953T > C
8	F	29	18	l	0.5	11	c.-32-13T > G	c.877G > A
9	M	38	21	l, a, r, s	0.5	11	c.-32-13T > G	c.1051delG
10	F	64	47	l	3	12	c.-32-13T > G	c.271G > A
11	M	62	24	l, a, s	34	3	c.-32-13T > G	c.1051delG
12	F	50	nk	l, a, r	nk	8	c.-32-13T > G	c.271delG
13	M	39	nk	l, a, r	nk	*	c.-32-13T > G	c.955 + 2T > G
14	M	37	28	l, a, s	8	1	c.-32-13T > G	c.271 G > A
15	M	16	3.5	l, a	1.5	11	c.1076-22T > G	c.525delT
16	M	13	6	l, a, s	6.5	1	c.1548G > A	**c.1470C** **>** **A**
17	F	69	40	l, a, r	28	**	c.-32-13T > G	**c.323G** **>** **C**
18	F	25	nk	l, a	nk	**	c.-32-13T > G	c.2608C > T
19	M	15	15	HyperCK	0.5	0	c.-32-13T > G	**c.2380dupC**
20	M	11	8	HyperCK	3	0	c.-32-13T > G	c.2051C > G
21	W	13	12.5	HyperCK	0.5	0	c.1134C > G	c.1478C > T

New mutations are bold

*nk* not known, *l* limb-girdle weakness, *a* axial weakness, *r* respiratory weakness, *s* scapula alata, *HyperCK* asymptomatic hyperCKemia, *ERT* enzyme replacement therapy

* pt. decided to stop treatment after 2 years due to lack of efficacy and severe disease, ** ERT recently started

Two male pts (ID 19, 20) and one female pt (ID 21) were investigated because of pauci- or asymptomatic hyperCKemia (726 and 1447 U/l) at the age of 15, 11 and 13 years, respectively. Symptom onset in the other 18 LOPD patients (9 f/9 m) was 24.9 ± 13.9 years (range: 3.5–47 years). At the initial evaluation, limb-girdle weakness was found in 17 patients; 15 of these also had axial weakness, 8 also had respiratory weakness and only 4 scapular winging. Isolated respiratory weakness was observed in only one case. Weakness was found in the proximal lower limbs in 17 cases, while the upper limbs were affected in only eight.

CK was elevated in all but one symptomatic pt; 990 ± 684 U/l (118–2437). EMG studies were available from 15 pts and fibrillations/positive sharp waves were documented in six cases, myotonic discharges in only two. MRI was performed in only four patients; a hypoplastic vertebral artery was found in two, while the MRI was normal in the others. All LOPD patients were compound heterozygous and the common Caucasian mutation c.-32-13T > G was found in 17 cases, new mutations in four (http://cluster15.erasmusmc.nl/klgn/pompe/mutations.html).

Diagnostic delay in the non-familial cases (*n* = 19) was 7.4 ± 9.7 years (range: 0.5–34 years). All but the pauci- or asymptomatic patients and one symptomatic patient, who declined treatment, are currently receiving ERT. Treatment was started just recently in two patients; the other 15 pts have been treated for 7.8 ± 3.5 years (range: 1–12 years). Treatment was well tolerated without side effects. Anti-rhGAA antibodies were tested in 7 pts and were positive in only one with a very low titre of 1:1600.

**Fig. 1:**
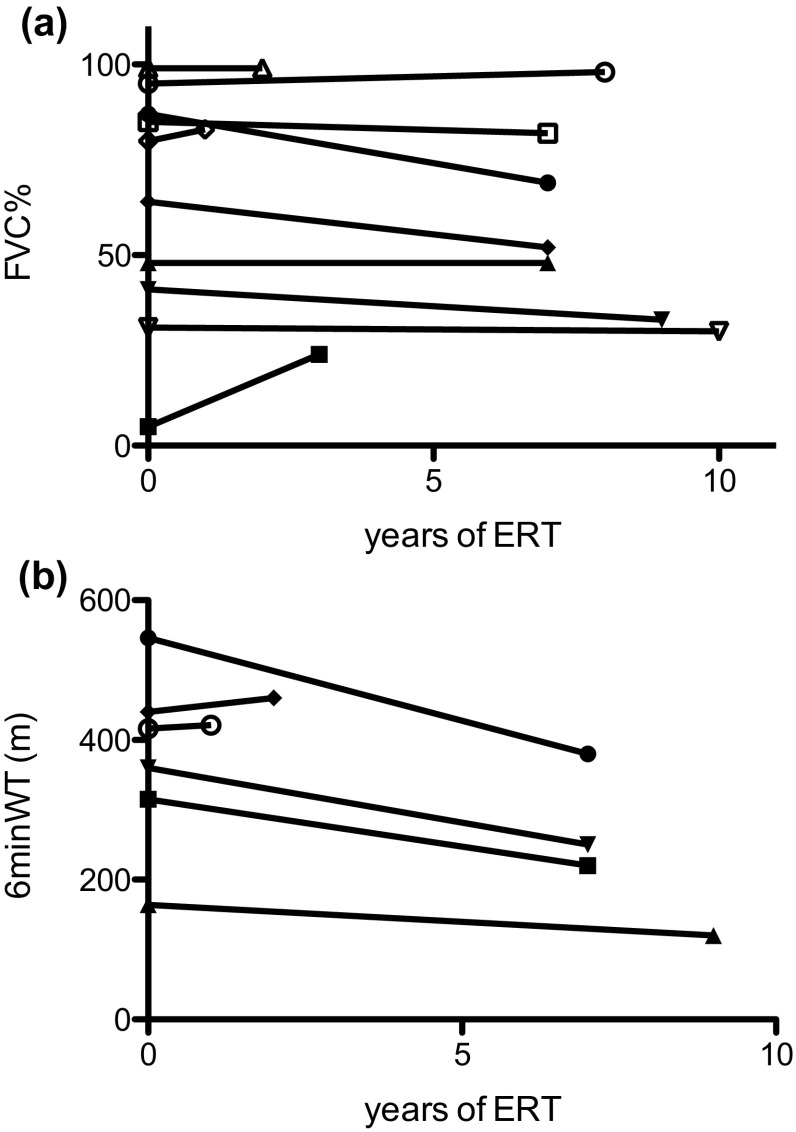
Effects of enzyme replacement therapy (ERT) on **a** FVC (*n* = 10) and **b** 6-min walk test (6minWT, *n* = 6)

The effect of ERT on 6minWT and FVC could be evaluated in only 6 and 10 pts, respectively (Fig. 1a, b). Mean FVC did not decline (before ERT: 63.6 ± 39.7%; last evaluation during ERT: 61.9 ± 26.9%; *P* = 0.5) while there was a trend towards a decline in the mean distance covered by the 6minWT (before ERT: 373.5 ± 117.9 m; last evaluation during ERT: 308.5 ± 120.8 m; *P* = 0.077).

## Discussion

The prevalence of Pompe disease in Austria found in the present study, 1:350,914, is considerably lower than that reported for Europe, 1:283,000 [4]. Although the prevalence of Pompe disease varies [2] it is reasonable to assume that there are still some undiagnosed individuals with Pompe disease in Austria. In the paediatric cohort, children with the severe, classical course of IOPD are generally less prone to be missed due to the rapidly progressive, life-threatening course. In contrast, late-onset patients with slowly progressive myopathy but without cardiac involvement experience not only a significant diagnostic delay but the diagnosis may also be missed.

The frequency of undiagnosed LOPD has been investigated in several studies [6, 12–17] and in the largest of these studies, 2.4% of patients with hyperCKemia or limb-girdle muscular dystrophy (LGMD) of unknown origin were found to have LOPD [6]. This suggests that there are also some undiagnosed LOPD patients in Austria, and is supported by pts 19, 20 and 21, who were diagnosed by whole exome sequencing for diagnostic workup of pauci- or asymptomatic hyperCKemia.

The clinical symptoms of the Austrian LOPD cohort are in line with the typical findings in Pompe disease [18–20]. Core features are proximal lower limb and axial weakness, frequently in combination with respiratory weakness, while scapular winging is rare. Although such a pattern of weakness is also seen in LGMD, especially prominent axial and respiratory weakness should alert the clinician to search for Pompe disease. Despite these suggestive clinical features, mean diagnostic delay was 8.8 years, which is similar to previous reports [18–21]. Several factors have been found to influence the diagnostic delay, such as presenting signs and symptoms, year of diagnosis and age at symptom onset [18–21]. Due to the small sample size, these factors cannot be statistically analysed in our population, but it seems that age is an important factor as the mean diagnostic delay was 18.6 years in patients currently older than 40 years and only 2.8 years in patients younger than 40 years. As Pompe disease is a progressive disorder, which causes significant morbidity and increased mortality [7], early diagnosis and treatment are essential [22]. It remains to be seen whether simplified diagnostic procedures such as the dried blood spot test [23], easily accessible genetic testing and campaigns to increase awareness will further reduce the diagnostic delay in the near future.

Besides typical clinical findings, electromyographic (EMG) abnormalities, in particular spontaneous activity and myotonic discharges, have been considered to be suspicious for Pompe disease [24]. Results from EMG studies were available in only 15 of our patients and myotonic discharges and abnormal spontaneous activity were observed in only two and six patients, respectively. Only a few studies reported the frequency of these EMG abnormalities in Pompe disease, and some found them frequently [25, 26] others only rarely [27, 28] in their population. This discrepancy might be explained by the muscles studied as Kassardjian et al. [26] clearly showed that the highest diagnostic yield can be achieved when sampling from paraspinal muscles and the tensor fasciae latae muscles. This might also explain the low rate of EMG abnormalities in our cohort as these muscles have not been investigated.

The c.-32-13T > G mutation was the most frequent mutation (85%) found in Austrian LOPD patients, which equals reports from nearby European countries [19, 27–30]. Similar to other studies, we also found some new mutations, which were all predicted to be pathological and Pompe disease has been verified by reduced enzyme activity either in the dried blood spot test or in muscle tissue. In contrast, the c.-32-13T > G mutation was only seen in 50% of IOPD. The known mutations found in IOPD are predicted to be severe with the exception of c.784G > A, which is predicted to be potentially less severe (http://cluster15.erasmusmc.nl/klgn/pompe/mutations.html). In this case the second, yet undescribed frameshift mutation c.1057delC, p.(Gln353Serfs*39) probably defines the rather severe phenotype.

The response to ERT could be evaluated in only about half of the patients who underwent yearly standardized assessments. Recently published European guidelines [11] recommend a standardized regular monitoring to gain more knowledge about the disease course under ERT to facilitate informed, cost-sensitive treatment decisions. As can be seen from the figures, the effects of ERT vary between patients and as nicely described by [22] there are obvious responders and non-responders. In the patients we were able to analyse, the 6minWT improved or remained unchanged in only two of six, while FVC improved or remained unchanged in seven of ten. This is somewhat different to treatment responses reported in a large meta-analysis [7]. However, a comparison between the published results and those presented here seems invalid, as our population is small and the observation periods vary greatly.

To summarize, in this study we report the phenotypes and genotypes of Austrian IOPD and LOPD patients and their response to treatment. The genotype corresponds to that described in other European countries and the reported clinical features corroborate the typical phenotype in LOPD with limb-girdle and axial weakness in the majority of patients. The incidence in Austria, however, is lower than that reported for Europe, suggesting that some patients go undiagnosed. Despite the fact that all patients have access to ERT, the recommended yearly standardized assessment is only performed in about half.
